# Functional Response of a Near-Surface Soil Microbial Community to a Simulated Underground CO_2_ Storage Leak

**DOI:** 10.1371/journal.pone.0081742

**Published:** 2013-11-26

**Authors:** Sergio E. Morales, William E. Holben

**Affiliations:** 1 Department of Microbiology and Immunology, Otago School of Medical Sciences, University of Otago, Dunedin, New Zealand; 2 Cellular, Molecular and Microbial Biology Program and Systems Ecology Program, Division of Biological Sciences, The University of Montana, Missoula, Montana, United States of America; 3 Montana—Ecology of Infectious Diseases Program, The University of Montana, Missoula, Montana, United States of America; Missouri University of Science and Technology, United States of America

## Abstract

Understanding the impacts of leaks from geologic carbon sequestration, also known as carbon capture and storage, is key to developing effective strategies for carbon dioxide (CO_2_) emissions management and mitigation of potential negative effects. Here, we provide the first report on the potential effects of leaks from carbon capture and storage sites on microbial functional groups in surface and near-surface soils. Using a simulated subsurface CO_2_ storage leak scenario, we demonstrate how CO_2_ flow upward through the soil column altered both the abundance (DNA) and activity (mRNA) of microbial functional groups mediating carbon and nitrogen transformations. These microbial responses were found to be seasonally dependent and correlated to shifts in atmospheric conditions. While both DNA and mRNA levels were affected by elevated CO_2_, they did not react equally, suggesting two separate mechanisms for soil microbial community response to high CO_2_ levels. The results did not always agree with previous studies on elevated atmospheric (rather than subsurface) CO_2_ using FACE (Free-Air CO_2_ Enrichment) systems, suggesting that microbial community response to CO_2_ seepage from the subsurface might differ from its response to atmospheric CO_2_ increases.

## Introduction

Carbon capture and storage (CCS) technology is being explored as an option for reducing carbon dioxide (CO_2_) emissions to the atmosphere [1-3]. For this option to achieve significant reductions in CO_2_ emissions, approximately 1 teraton (one trillion tons) of atmospheric CO_2_ will have to be removed and/or prevented from entering the atmosphere [1]. Under current CCS scenarios geologic carbon sequestration requires injecting supercritical CO_2_ deep in the subsurface (>1 km) into porous sedimentary formations in natural sites such as deep geological cavities, saline aquifers, spent oil or gas fields, coal mines or on the ocean floor [1-3]. However, supercritical CO_2_ expands and tends to be buoyant, continuously moving upwards until a “cap rock” (e.g. a barrier of non-porous rock) is encountered. This fact, and the ability of a CO_2_ plume to spread out over a large area suggest that, over time, pathways for CO_2_ to move upward and escape could lead to stored gas reaching surface soils or the atmosphere [3]. This has led to research aimed at developing monitoring techniques for the detection of CO_2_ leaks from geologic formations, and to studies on the effects such leaks could have on surface ecosystems [4-6]. 

Thus far, examples of the effects underground CO_2_ leaks can have on surface ecosystems are primarily limited to large natural magmatic CO_2_ emissions as reported at Mammoth Mountain, California [7,8] and a volcanic vent in Germany [9]. One controlled-release CO_2_ research site is the ASGARD site located on a grassland at the University of Nottingham’s Sutton Bonington Campus, which comprises permanently installed underground pipework emitting CO_2_ at a rate of 3 liters per minute at a depth of between 50 - 60 cm below ground surface [10]. This flow rate represents the equivalent of 3 tons y^-1^, or 0.0003% of what is injected into an active storage site [11]. A 19-week (May to September 2006) gas release at this site resulted in changes in plant coverage and a significant drop in bacterial abundance in areas of high CO_2_ concentration, with microbial activity (measured as ATP concentration) also decreasing (in some cases to below detection) in response to high (87%) CO_2_ levels [10]. To date, though, the majority of studies of elevated CO_2_ effects on microbial, plant and animal communities have focused on the effect of elevated atmospheric CO_2_ as conducted in FACE (free-air CO_2_ enrichment) sites or similar scenarios. Thus biological response in CCS leak scenarios is lacking. 

Here, we report the effect of a simulated underground CO_2_ leak (e.g. as in escape from a deep subsurface CO_2_ sequestration reservoir) on microbial populations at a shallow subsurface controlled-release facility in Bozeman, Montana, USA. During 2009, two separate controlled releases (spring and summer) of CO_2_ were carried out at the Zero Emission Research and Technology (ZERT) field site. Our objectives were to determine: (1) the effect of CO_2_ seepage on microbial communities in near-surface soils; (2) how microbial response was affected by seasonal variations; and (3) the suitability of monitoring the microbial community and its constituent guilds as sentinels for elevated CO_2_ levels. 

## Materials and Methods

### Study Site, CO_2_ Release Regime and Sample Collection

The ZERT field site (both control and CO_2_ exposed areas) and its shallow subsurface controlled release facility have been previously described in detail [12-15]. The field studies did not involve endangered or protected species and no specific permissions were required for sample collection this location as it is part of the university facilities. In brief, the site is an agricultural plot located at the Montana Agricultural Experiment Research Center, at Montana State University in Bozeman MT, dominated by mixed grasses (70%) with the remainder being alfalfa (15%), clover (8%), dandelion (5%) and various forbs (2%) [6]. The whole field site consists of plants that were there previously (or descendants of those plants) when the field was used as a pasture, and vegetation is consistent between the control and CO_2_ sparged areas (M. Apple, personal communication). The soil structure throughout the field site (control and treatment sites) is characterized by a ~30 cm-thick clay topsoil overlying a ~20 cm-thick clayey silt layer, which overlies an alluvial sandy cobble with 10-25 cm diameter cobbles [15]. 

The infrastructure (i.e. ‘plumbing’) for subsurface CO_2_ release is described in full elsewhere [13,14]. In brief, a 98 m long horizontal well (304L stainless steel pipe) is located 2 m below the ground surface. The central 70 m are slotted to allow CO_2_ to vent into the soil. Six separate zones, spaced 10 m apart, are plumbed separately allowing for flow rates to individual zones to be varied independently. 

Previous to the experiment described herein, three prior separate releases had been carried out in 2007 and 2008. From 9–18 July 2007 (Release 1) and from 3–10 August 2007 (Release 2), 0.1 t CO_2_ day^-1^ and 0.3 t CO_2_ day^-1^ were released, respectively. From 9 July to 7 August 2008 (Release 3) 0.3 t CO_2_ day^-1^ were released. For the current experiment, two separate releases were carried out. The spring release (hereafter ‘June release’) began June 7, 2009 and lasted until June 14. The sustained release rate was 0.3 t CO_2_ day^-1^. A second summer release (hereafter ‘July release’) of 0.4 t CO_2_ day^-1^ occurred from July 17 through August 4, 2009. 

Soil sampling for our experiments was performed between June 6 and September 9, 2009 (spanning the spring to late summer seasons) by collecting soil cores consisting of the top 5 cm of soils (2 cm dia. X 5 cm deep) directly above the horizontal well, at adjacent points roughly in the center of Zone 2 [13,14]. Care was taken to collect cores between plants and to minimize inadvertent collection of roots or other tissues, with any visible contaminating plant materials removed from the soil with forceps prior to nucleic acid recovery. At each time point, a CO_2_-negative control sample was taken from an adjacent plot approximately 75 m northeast of the experimental site. This location was chosen as it is predominantly upwind from the CO_2_ release zone in an unused and unimpacted area of the site. Both sampling locations (control and treatment) are within a pasture site actively managed for feed hay, with harvesting occurring once a year. An aerial view of the site and detailed description of soil and plant communities can be found here: [6,16]. Samples were collected every 6 hours for 24 hours prior to CO_2_ releases, and continued at 6-hour intervals for 24 hours post-release. Multiple additional samplings were performed in the weeks following and between CO_2_ releases. All soil samples were stored in Whirl-Pak bags on dry ice in the field and kept at -70°C until processed for microbial community nucleic acid extraction.

### Environmental Conditions

Atmospheric temperature, relative humidity and barometric pressure were monitored continuously on-site by the ZERT Weather Station (http://orsl.eps.montana.edu/weather/zert/).

### DNA & RNA extraction and real-time quantitative PCR assays

Total community DNA and RNA were extracted from 1 g of each manually homogenized soil sample, in triplicate using the MoBio PowerSoil™ Total RNA Isolation Kit (MoBio, Solana Beach, CA) in conjunction with the MoBio RNA PowerSoil® DNA Elution Accessory Kit according to the manufacturer’s instructions. All purified DNA samples were stored at -20°C until used in downstream analyses. All RNA samples were immediately treated with RQ1 RNase-Free DNase (Promega, Madison, WI) and assessed for integrity using denaturing agarose gels and standard protocols. A subset of DNase treated samples were used as negative controls for RT reactions to confirm complete degradation of DNA. All purified RNA samples were stored at -70°C until used in downstream analyses. 

Real-time qPCR was performed using modified conditions from [17]. Reactions were performed in an iCycler iQ thermocycler (Bio-Rad, Hercules, CA) with an ABsolute Blue qPCR Sybr green ROX mix (ABgene, Rochester, NY) using primers and conditions summarized in Table S1. Each qPCR run included relevant known template standards made from cloned PCR products as described previously [17] and summarized in Table S1. Reaction mixtures (25µl) included 1µl of template (see concentration below), 10 pmol of each primer, 20µg bovine serum albumin, and 12.5 µl of ABsolute Blue QPCR Sybr green ROX mix. Specific genes and their respective transcripts targeted in this study were: *nifH*, nitrogenase gene; *nosZ*, nitrous oxide reductase gene; *nirS*, nitrite reductase gene; *rbcL*, ribulose-1,5-bisphosphate carboxylase oxygenase (RuBisCO) gene; *mxaF*, methanol dehydrogenase gene; and *mcrA*, methyl-coenzyme M reductase gene. In detail, the *nifH* gene was amplified using an initial denaturation step of 15 min at 95°C; followed by 10 cycles of touchdown consisting of denaturation (1 min at 96°C), primer annealing (1 min starting at 65°C and lowering 1.5°C per cycle), and primer extension (1 min at 72°C); followed by 30 cycles of amplification consisting of denaturation (1 min at 96°C), primer annealing (1 min at 50°C), and primer extension (1 min at 72°C), with a final extension step of 10 min at 72°C. The *nirS* and *nosZ* genes were amplified using an initial denaturation step of 15 min at 95°C; followed by 6 cycles of touchdown consisting of denaturation (15 s at 96°C), primer annealing (30 s starting at 63°C and lowering 1°C per cycle), and primer extension (15 s at 72°C); followed by 35 cycles of amplification consisting of denaturation (15 s at 96°C), primer annealing (30 s at 58°C), and primer extension (15 s at 72°C). The *mxaF* gene was amplified following the conditions for the *nifH* gene with a touchdown temperature starting at 65°C and lowering 1°C per cycle, and primer annealing for the remaining 35 cycles carried out at 55°C. The *mcrA* gene was amplified using an initial denaturation step of 15 min at 95°C; followed by 45 cycles of amplification consisting of denaturation (40 s at 96°C), primer annealing (1.5 min at 55°C), and primer extension (2 min at 72°C), and a final extension step of 10 min at 72°C. The *rbcL* gene was amplified using the same conditions as the *mxaF* gene but with a touchdown temperature starting at 65°C and lowering 1°C per cycle, and primer annealing for the remaining 35 cycles carried out at 55°C. Standards were constructed, and target specificity was assessed for each target by PCR amplifying DNA extracted from the field site. PCR products were then cloned and sequenced as described previously [18] to confirm specificity of each assay.

Gene abundance measurements were determined by quantifying specific gene copy numbers in 5 ng of total community DNA from individual replicate extractions. For measurement of specific gene transcript abundances, 0.525µg of RNA was reverse transcribed in a 35µl reaction using random hexamer primers (Promega) and the Omniscript Reverse Transcriptase (Qiagen, Valencia, CA) according to the manufacturer’s recommended protocol. Subsequently, 1µl of RT reaction product was used directly for real-time qPCR of gene transcripts. All qPCR reactions for any single sample were run at least in triplicate (for each individual extraction) as described above. 

### Statistical analysis

Relationships between ambient atmospheric conditions (i.e. weather parameters) and microbial gene and transcript abundances were determined by principal components analysis (PCA) with data matrices composed of environmental data and mean values for qPCR data. Data was organized with rows representing different sampling dates and columns representing individual variables. Principal component scores were plotted for the first two principal components. Parameters driving the ordination of the PCA plots were determined by querying all variables against the first two principal components. 

To account for variance associated with replicate sampling and technical replication, all qPCR data was analyzed independently. Individual variables were independently queried to each other by conducting pair-wise correlations and the non-parametric Spearman’s ρ. One-way analysis of variance (ANOVA) and the non-parametric Wilcoxon method were used to test for significant CO_2_ effects on the various microbial parameters. 

## Results

### Microbial functional response to CO_2_ exposure

**Figure 1:**
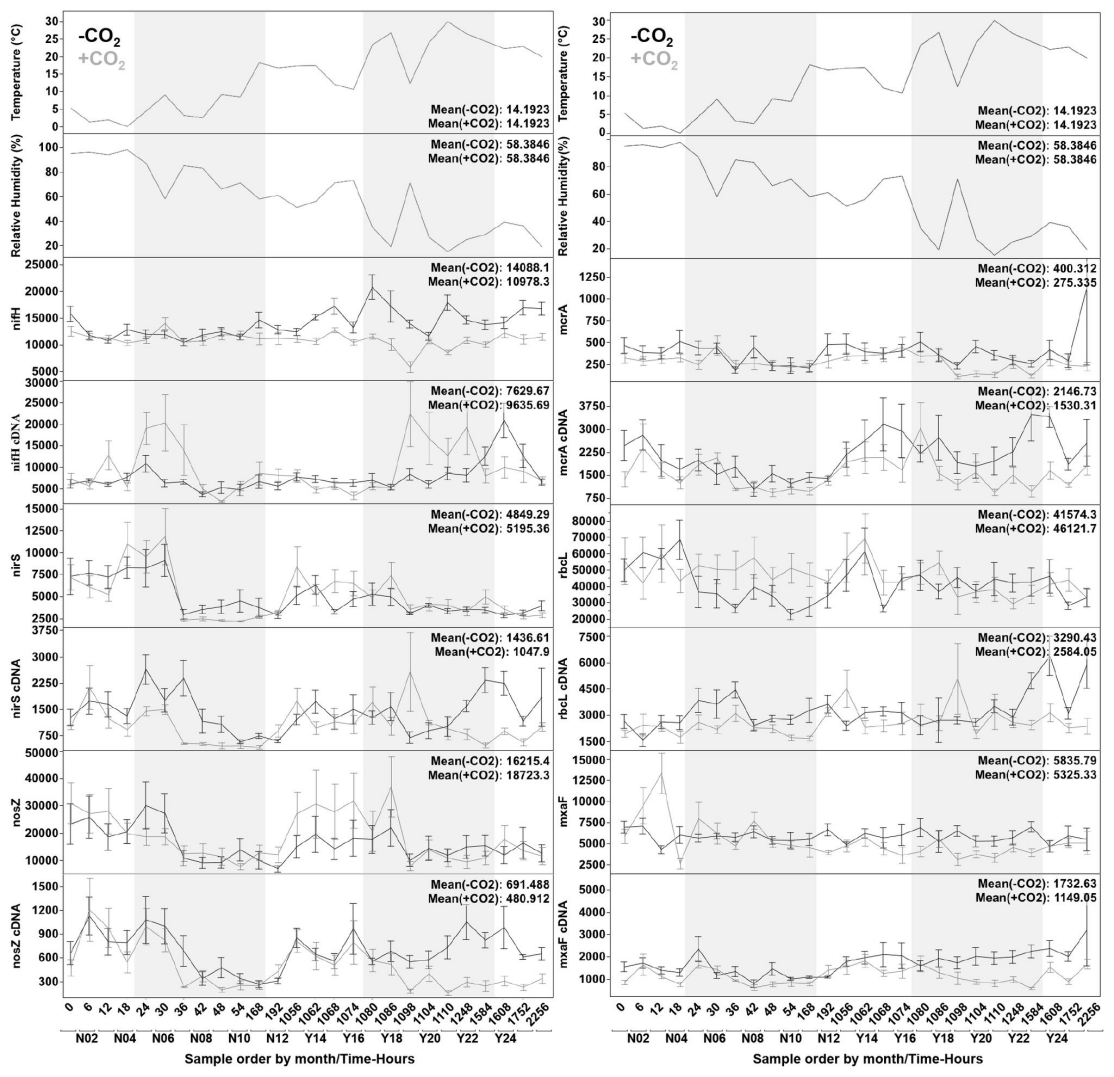
Community response to CO_2_ exposure over time. Plot of atmospheric temperature, relative humidity, gene abundance and transcript abundance at time of sampling for six functional genes involved in nitrogen (left panel) and carbon (right panel) cycling under simulated CO_2_ leakage (grey lines) or background (black lines) conditions. Data is presented sequentially by month (N=June, Y=July-Sept) and by hours from start of the experiment. Grey-shaded bars indicate active CO_2_ flux (injections) for June and July. Values indicate copy numbers per 5 ng of nucleic acid based on averaged measurements of separate triplicate extractions and at least triplicate qPCR reactions for each extraction (n≥9). Error bars are one standard error of the mean (SE). *nifH*: nitrogenase gene; *nosZ*: nitrous oxide reductase gene; *nirS*: nitrite reductase gene; *rbcL*: ribulose-1,5-bisphosphate carboxylase oxygenase (RuBisCO); *mxaF*: methanol dehydrogenase; *mcrA*: methyl-coenzyme M reductase.

To determine community dynamics in response to CO_2_, we compared functional gene (DNA) and transcript (mRNA) abundance for all six functional gene targets over time for both control and experimental samples (Figure 1). These targets were chosen as they provide a good overview of the prokaryotic organisms controlling net inputs and outputs of C and N from soils, as well as the chemical state of any losses (e.g. whether carbon was lost as CO_2_ or CH_4_, and nitrogen as N_2_O or N_2_). Results are summarized in Table 1, with details of the statistical analyses presented in Table S2. No consistent pattern emerged across all six targets, but both gene and transcript abundances for each target were affected in some way. The number of nitrogen fixers, as determined by abundance of the nitrogenase gene (*nifH*), decreased in response to elevated CO_2_. This decrease was temperature dependent with 1.3 to 1.6-fold mean differences during and following the July release. However, the activity of nitrogen fixers, as determined by the abundance of *nifH* gene transcripts did not respond similarly. The *nifH* transcript abundance values showed up to a 1.7-fold mean increase in response to CO_2_ release, with a subsequent 1.3-fold decrease upon cessation of CO_2_ release. 

**Table 1:** Fold changes in abundance (means) in response to CO_2_ release based on sampling time.

	**DNA**					**mRNA**				
**Target**	**Pre-injection**	**Injection (June)**	**Injection (July)**	**Injection (All)**	**Post-injection**	**Pre-injection**	**Injection (June)**	**Injection (July)**	**Injection (All)**	**Post-injection**
***nifH***	**-1.1(0.03;0.10**)	1.0(0.28;0.39)	**-1.6(0.00;0.00**)*	**-1.3(0.00;0.00**)*	**-1.3(0.00;0.00**)*	+1.2(0.30;0.16)	**+1.7(0.01;1.00**)	**+1.6(0.01;0.86**)	**+1.6(0.00;0.90**)	**-1.3(0.04;0.04**)*
***nirS***	1.0(0.87;0.29)	-1.1(0.68;0.35)	+1.2(0.12;0.41)	1.0(0.73;0.21)	+1.2(0.09;0.86)	-1.1(0.49;0.20)	**-1.9(0.00;0.00**)*	**-1.1(0.49;0.03**)	**-1.5(0.00;0.00**)*	**-1.4(0.01;0.07**)
***nosZ***	+1.2(0.31;0.18)	-1.2(0.34;0.07)	1.0(0.86;0.30)	-1.1(0.64;0.72)	+1.5(0.03;0.04)*	**1.0(0.05;0.10)**	-1.4(0.06;0.31)	**-2.2(0.00;0.00**)*	**-1.7(0.00;0.00**)*	**-1.4(0.02;0.00**)*
***mcrA***	**-1.4(0.02;0.13**)	-1.2(0.24;0.30)	**-1.7(0.00;0.00**)*	**-1.4(0.00;0.00**)*	-1.5(0.13;0.18)	**-1.4(0.05;0.06**)	-1.2(0.11;0.11)	**-1.5(0.00;0.00**)*	**-1.4(0.00;0.00**)*	**-1.5(0.00;0.01**)*
***rbcL***	-1.2(0.27;0.09)	+1.6(0.00;0.00)*	-1.1(0.49;0.38)	+1.2(0.01;0.01)*	+1.1(0.22;0.08)	-1.1(0.33;0.21)	**-1.5(0.00;0.00**)*	-1.1(0.60;0.08)	**-1.2(0.01;0.00**)*	**-1.4(0.00;0.00**)*
***mxaF***	+1.3(0.07;0.47)	1.0(0.74;0.59)	**-1.5(0.00;0.00**)*	**-1.2(0.00;0.00**)*	**-1.2(0.01;0.01**)*	**-1.4(0.03;0.00**)*	**-1.3(0.01;0.04**)*	**-1.9(0.00;0.00**)*	**-1.6(0.00;0.00**)*	**-1.4(0.01;0.01**)*

P-values based on ANOVA and non-parametric Wilcoxon methods are shown in parenthesis respectively. Bold cells indicate significantly different results (P-value ≤ 0.05) for at least one of the two methods. Significantly different results using both methods are denoted with an asterisk (*).

Nitrite reducers, as determined by abundance of the nitrite reductase gene (*nirS*), also displayed a temperature dependent decrease. This decrease was restricted to the active release period in June when a short drop in temperature was recorded; however no statistically significant change in total mean was detected for that period. A similar pattern was seen with nitrite reducing activity, where a 1.9-fold decrease in transcript abundance was observed during the June release. As the temperature dropped again late into the second release, an effect was once again observed, with a statistically significant difference detected between the total control and experimental means. 

Nitrous oxide reducers, as indicated by abundance of the nitrous oxide reductase gene (*nosZ*), were slightly increased after the first release, but the effect was not sustained. In contrast, the activity of the nitrous oxide reducers showed a similar response to that of the nitrite reducers, with an early decrease in transcript abundance during the June release. However, nitrous oxide reducer activity showed a strong response during the July release, with a 1.4 to 2.2-fold decrease in transcript abundance sustained until 28 days after cessation of CO_2_ release.

Autotrophic bacterial numbers, as indicated by abundance of the ribulose-1, 5-bisphosphate carboxylase oxygenase (RuBisCO) gene (*rbcL*), showed a 1.6-fold increase during the June release, but no significant effects were noted at any other time. RuBisCO gene transcript abundance, however, indicated a 1.5-fold decrease in response to the June release and also a sustained 2-fold decrease starting late during the July release, which continued until the end of the experiment. 

Methanogen numbers, as determined by abundance of the methyl-coenzyme M reductase gene (*mcrA*), showed a 1.4-fold decrease prior to the June CO_2_ exposure. Although numbers were generally lower in treatment plots, a significant decrease (1.7-fold) was not seen until the July release, and those numbers recovered after cessation of the release. Methanogen activity response closely followed methanogen abundance, with a 1.5-fold decrease in transcript abundance in response to the July release and recovery not observed until 28 days later.

Methanotroph numbers, as determined by abundance of the methanol dehydrogenase gene (*mxaF*), decreased 1.5-fold, with post injection numbers following the June injection driving the significant decrease observed. A decrease in transcript abundance was also detected, with 1.3 to 1.9-fold decreases throughout the entire experiment.

### Multivariate analysis of microbial response to CO_2_ exposure

**Figure 2:**
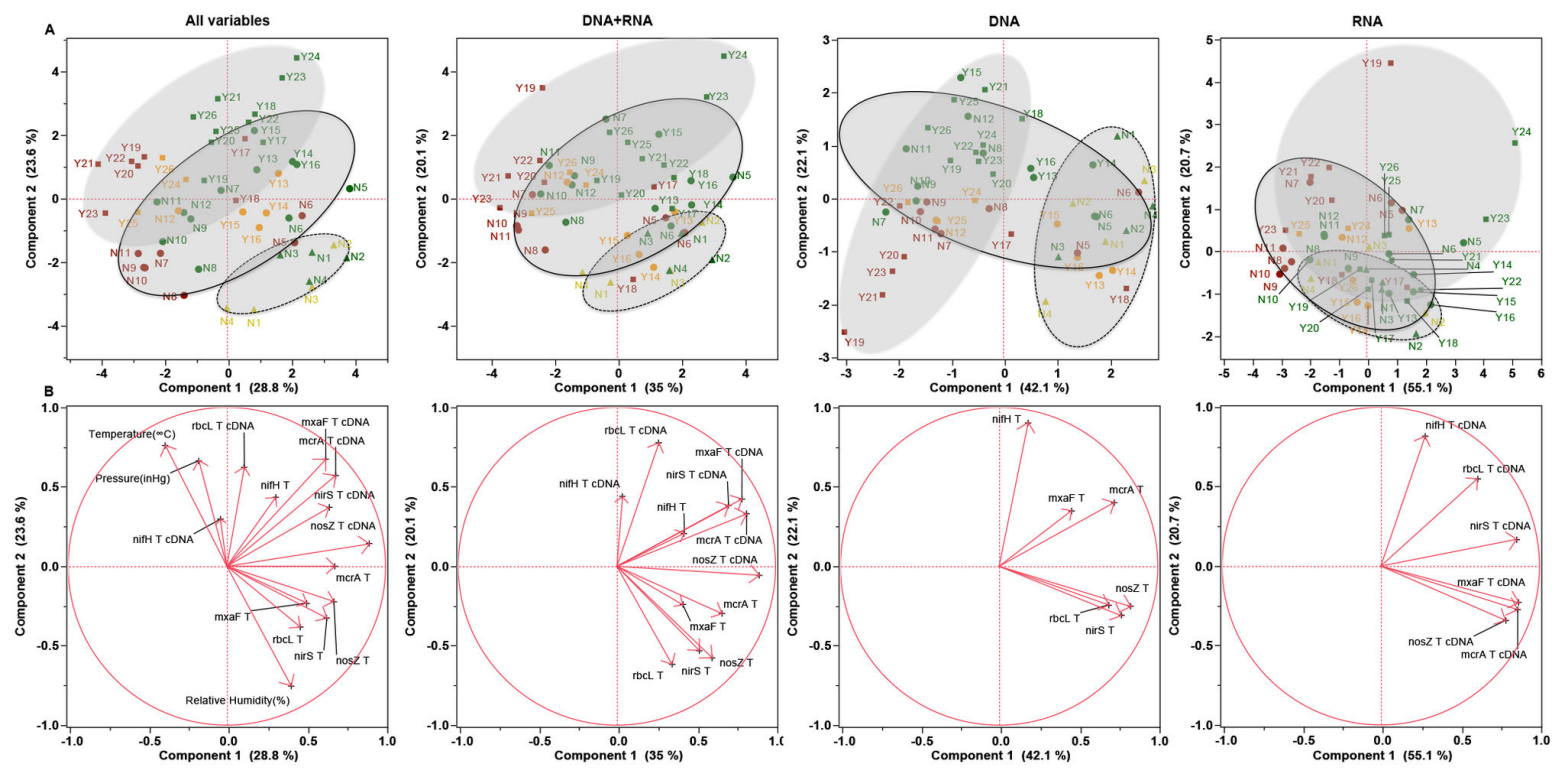
Principal component analysis (A) and factor loadings (B) for (from right to left) transcript (mRNA) abundances, gene abundances, gene and transcript abundance combined, and gene and transcript abundances combined with atmospheric data. Samples are labeled sequentially by treatment (green=background site; red=treatment site active CO_2_ injection; orange=treatment site post-CO_2_ injection; and yellow=treatment site pre-CO_2_ injection), and by month (N=June, Y=July-Sept). Shaded areas represent pre-injection period (N1 - 4, ▲), June injection period (N5 - 12 and Y1 3- 16, ●) and July injection period (Y17 - 26, ■). The percentage of the variation in the samples described by the plotted principle components is indicated on the axis. Gene names in Panel B are as for Figure 1.

Principal components analysis (PCA) on mean values revealed both a seasonal response as well as a CO_2_ effect on gene and transcript abundance (Figure 2 and Figure S1). Both mRNA and DNA showed marked responses, although clustering based on DNA was more defined. PCA based solely on gene abundance, transcript abundance, or all variables combined showed clustering based on seasons, with the CO_2_ effect being strongest during active CO_2_ release. Post-injection samples (between the June and July release, and after the July release) displayed some recovery, with CO_2_-exposed samples shifting away from active release samples and toward background controls. In this analysis, 75.8% of the variance could be explained by the first two components in a PCA with only transcript data, with an additional 10.5% explained by the third component (86.3% total). The percent of variance accounted for in the first three components decreased when PCAs were performed with just the gene abundances (79.6%), with gene and transcript abundance combined (68.4%), or for all abundance data and atmospheric data (64.1%). A factor analysis (Table S3) for each individual PCA suggested that clustering was driven on one axis by the CO_2_ effect and on the other by seasons. 

**Figure 3:**
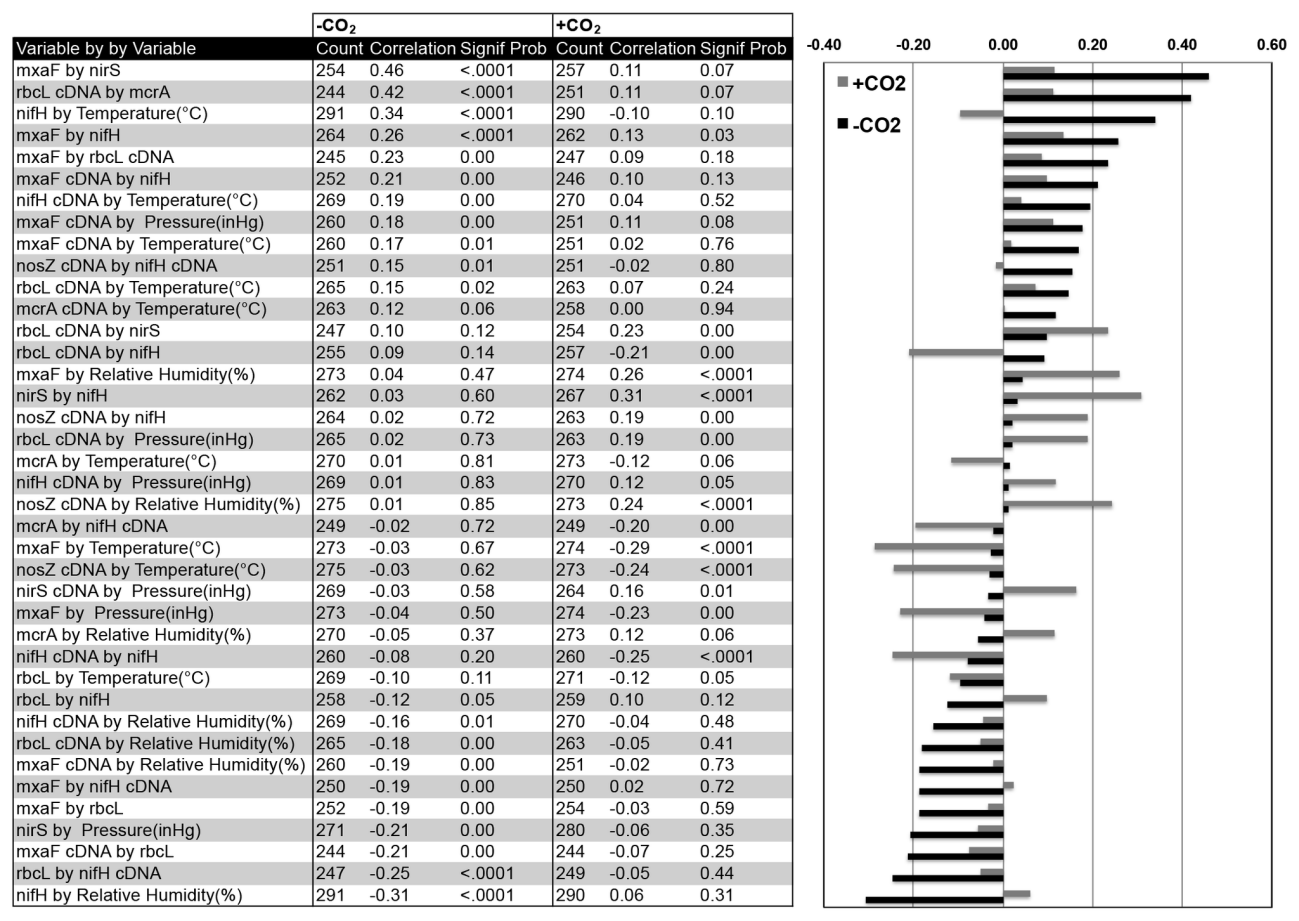
Changes in pairwise correlation between different variables in response to CO_2_. All data points for all variables were compared independently for background and CO_2_ exposed samples. Only variables displaying shifts in correlations in response to CO_2_ are presented. Non-parametric version of this figure can be found in Table S4, with full statistical results in Tables S5 and S6.

To account for changes in microbial community structure and activity relationships in response to CO_2_, pairwise correlations were performed on all data for background controls and CO_2_-exposed samples separately (summary of significantly affected correlations presented in Figure 3 and Table S4, with full statistical results in Tables S5 and S6 respectively). CO_2_ exposure resulted in loss of correlations naturally found in the background samples, while it created others not detected under normal conditions. However, these shifts were only found to occur in weakly correlated variables, and the new correlations that evolved as a result of CO_2_ exposure tended to be weak (<0.35 correlated) independent of the analytical methods used. 

## Discussion

In recent decades, CO_2_ has led the trend of increasing greenhouse gases in the atmosphere with a 28% increase in CO_2_ emissions between 1990-2004 [19]. In 2004, CO_2_ emissions were estimated at 30 Gt CO_2_ equivalents/year, with emissions projected to increase 40% to 110% by 2030 [19]. The Intergovernmental Panel on Climate Change has identified CCS as a potential mitigation strategy. In this scenario, storage methods could include injection of CO_2_ into underground geological formations, into the deep-ocean, or even industrial fixation as inorganic carbonates. In the case of underground storage, the continuous storage of such large amounts of CO_2_ will require assessment of the potential environmental impacts from accidental release of stored gases. Early studies have identified potential lethal effects on plants and subsoil animals and contamination of groundwater from unintended releases [3,4,6,20]. However, the effect of accidental CO_2_ release on the microbial populations and guilds driving biogeochemical cycles has not been studied. Here, we show that a short-term, low-rate, simulated underground leak significantly altered both the abundance and activity of key microbial populations responsible for carbon and nitrogen cycling in soils. 

The current study shows that seasonal patterns affect soil microbial communities at both DNA and RNA levels, even when exposed to elevated CO_2_ conditions, but that the specific seasonal response patterns were altered. This suggests that two different microbial community responses might be simultaneously at play in this system, namely ecological (i.e. environmental) selection on population/guild abundance and regulation of biogeochemical activities at the level of transcription. Elevated CO_2_ resulted in both positive and negative shifts of both DNA and mRNA levels of nitrogen and carbon cycling genes (Figure 1 and Table 1). In the case of the nitrogen fixer population, a 1.3 to 1.6-fold decrease in abundance was seen in response to elevated CO_2_, while mRNA levels were increased up to 1.7-fold. This suggests that only a portion of the original population was capable of surviving under high levels of CO_2_, but those that did persist tended to respond rapidly by increasing expression (via up-regulated gene transcription) of nitrogen fixation pathways. The opposite effect was observed for denitrifiers carrying out nitrous oxide reduction; in this case there was an increase in the population both pre- and post-injection. In contrast, although no difference was detected during injections in relationship to background samples, the abundance of *nosZ* genes was lower during CO_2_ injections in exposed samples compared to other CO_2_-exposed samples pre- and post-injection. This suggests that although no effect was seen between the treatments during the injection period, there was variance within the same treatment over time, which is not detected unless time series are examined. *nosZ* transcriptional activity during injections decreased up to 2.2-fold compared to background samples. Further, at this transcriptional level, the negative effect continued well past the July injection. The clearest example of these two response mechanisms in action is seen during the June injection for the *rbcL* (RuBisCO) targets. Although the CO_2_ pulse led to an increase in the number of organisms harboring this gene, the total level of transcription was equally reduced, indicating that although organisms capable of carrying out this reaction were present, they were not all up-regulating the transcription of the gene, and thus either a lower percentage of the autotrophic population was actively carrying out the function of fixing CO_2_ via RuBisCO or there was a generalized suppression of RuBisCO gene expression. The observed increase in abundance coincided with the plant-growing season. 

While studies on the effects of elevated CO_2_ in soils are not uncommon, most studies used ppm ranges of CO_2_ ranging between atmospheric (year-dependent, approximately 350 ppm) and a maximum of 1200 ppm to assess the environmental effects of elevated atmospheric CO_2_. In contrast, the current study used a below-ground delivery system that creates “hot zones” above leak points, indicating that gases tended to escape directly upwards [16,21] leading to concentrations that would be equivalent to 2,500-30,000 ppm within a “hot zone” [12,14] to emulate and assess impacts of escaped CO_2_ from a CCS site. Also, most studies have relied on elevated CO_2_ treatments which primarily affect the top 10 cm of soils as in FACE facilities [22], while our approach saturated the entire soil column above the injection zone and the proximal aboveground area. Plant stress at the site could be detected up to 7.5 m away from the release zone, with the most distinguishable plant stress signatures within 2.5 m [6,23]. This suggests that any response seen in previous studies using lower concentrations of CO_2_ could be magnified in a CCS escape scenario due to the much higher concentrations of CO_2_ involved. However, given that the pulses in our experiments were short-lived (1 - 4 weeks), maximal impacts from CO_2_ exposure may have not yet been manifested. 

We observed that, although exposure to CO_2_ altered the microbial community and its activity, it did not eliminate the seasonal response. In a previous study, Marhan et al. (2011) reported temporal variations, with decreases in denitrifiers linked to lower moisture levels during June - August, similar to what was observed in the current study, but they did not detect the CO_2_ effect that we describe [24]. Here, we observed that the first component of a PCA mostly accounted for the effect of CO_2_, while the second component accounted mostly for seasonal effects when all variables were examined (Figure 2 and Table S3). This, and the time-dependent (seasonally-dependent) response observed for many targets, indicates that CO_2_ effects are dependent on other factors. These other factors are not known for this site, but could include N and P levels in soils as suggested in prior studies with elevated atmospheric CO_2_ [22,25,26]. Although termination of subsurface CO_2_ injection apparently led to a start in recovery toward background levels of gene and transcript abundance, the speed of recovery was target-dependent and differences in pre-injection measures of these parameters suggest a legacy effect of elevated CO_2_ observable even 9 months after the prior injection. However a second possible interpretation is that high soil heterogeneity led to a simple discrepancy due to spatial variability, a factor that we cannot account for in this experiment. 

We also observed temperature/moisture-dependent responses to elevated CO_2_ as has been previously reported [22,27]. It has also been suggested that changes in precipitation tended to have a greater effect on microbial community composition than factors related to climate change [28], a phenomenon that was observed in a prior study during sharp temperature declines (data not shown). The ZERT field site also showed a plant-level response, with legumes and forb numbers decreasing under elevated CO_2_ and being replaced by short grasses [6]. This response differs from prior FACE experiments that have shown an increase in legumes, forbs, legume-bacterial symbiosis, nitrogen fixation genes and nitrogen fixation [29-31]. However other researchers have indicated that a positive response in nitrogen fixation is not universal, and is dependent on plant species and N and P availability [32-36]. 

In the current study the decline in nitrogen fixers was also accompanied by an increase in *nifH* transcriptional activity, suggesting that high levels of CO_2_ in soil might be selecting against symbiosis-based N fixation given that legume numbers were reduced, and towards a smaller, more CO_2_ tolerant, free living-nitrogen fixer community as suggested by Tissue et al [32]. Other microbial responses in the current study agree with previous work monitoring DNA-level changes, including increases in *rbcL* gene numbers and a marginal increase in *mcrA* gene numbers [31], although the variance accounted for in the current ZERT dataset is larger. The increase observed in *rbcL* (RuBisCO) gene abundance here was concomitant with decreased transcription. It has been noted that elevated levels of CO_2_ increase efficiency of RuBisCO in plants and algae [37]. If the same response is applicable to all carbon fixing microbes, it could mean that the excess CO_2_ is allowing a larger population of autotrophs to prosper, but due to a higher efficiency in CO_2_ assimilation they have reduced need for high-level expression of the *rbcL* gene. 

Two major findings in this study are that microbial community response is also elicited by environmental factors (moisture, temperature, nutrient, etc.) other than CO_2_, and that microbial response can be detected at both the population (DNA) level and the transcriptional level (mRNA), although these parameters do not always respond in the same way. Accounting for seasonally-dependent responses and contradicting responses at the DNA and mRNA level are important if we are to understand the effects of large CO_2_ leaks from geologic underground storage sites. Shifts in both functional populations and their activity, as reported herein, could create localized disruptions in ecosystem processes affecting soil biogeochemical cycles. These effects might be similar to those observed in previous studies using FACE systems, but due to the different CO_2_ delivery systems and gas concentrations used, additional assessments will be needed to determine the overall effect of leaks from underground storage. It is also important to note that the simulated leaks in these experiments represented low leakage rates for relatively short periods of time. Sustained exposure or higher CO_2_ levels in real-world storage leak scenarios might well result in different or more drastic responses. Since we cannot directly identify the mechanisms leading to the observed shifts, we can only speculate. Elevated levels of CO_2_ could be directly affecting these communities, or indirectly doing so by altering factors such as pH. However, soil pH measurements taken 5cm below the surface along a transect in 2009 (pre- and post-injections) showed no indication of a pH shift with an average of 6.65 (std. dev. = 0.38) (M. Apple, personal communication) so other factors including micro-scale variation could be involved [38].

Considering the current work in a broader context, a number of recent studies by our group and others show that microbial communities in general are highly dynamic, even under entirely ambient conditions. Thus, microbial responses to other perturbations such as elevated CO_2_ may be masked by, embedded within, or quenched by natural responses to ambient environmental change. This means that more intense short-term temporal and seasonal sampling of microbial communities under investigation should be performed in order to fully delineate, define and understand their response to perturbation. Further, in environments exhibiting heterogeneous distribution of microbial populations and their corresponding functions, sampling should be replicated commensurately to allow investigators to measure natural variance in microbial community responses, rather than averaging that variance through homogenization of large samples or bulking of multiple small samples. Finally, with increasing knowledge of microbial ecology, it becomes clear that microbes are not just responding to, but in many cases driving, changes that alter ecosystems [31,39]. Thus, continued studies that attempt to observe, understand and explain the integral role of microbes in both local and global biogeochemical cycles are necessary. 

## Supporting Information

Figure S1 **Principal component analysis for (from right to left) transcript (mRNA) abundances, gene (DNA) abundances, transcript and gene abundance combined, and gene and transcript abundances combined with atmospheric data.** Panel (A) shows June sample data only. Panel (B) shows only the July - Sept sample data. A compiled view for all samples can be seen in Figure 2. Samples are labeled sequentially by treatment (green=background site; red=treatment site active CO_2_ injection; orange=treatment site post-CO_2_ injection; and yellow=treatment site pre-CO_2_ injection), and by month (N=June, Y=July-Sept). The percentage of the variation in the samples described by the plotted principle components is indicated on the axis.
(TIF)

Table S1 **Primers and PCR conditions used in this study.**
(TIF)

Table S2 **Details for statistical analyses based comparison of target abundance in response to CO_2_ release based on sampling period.** Summary provided in Table 1.
(XLSX)

Table S3 **Factor analysis.**
(TIF)

Table S4 **Changes in non-parametric Spearman’s ϱ between different variables in response to CO_2_.**
(TIF)

Table S5 **Pairwise correlations for all variables with and without CO_2_ exposure.**
(XLSX)

Table S6 **Non-parametric Spearman’s correlations for all variables with and without CO_2_ exposure.**
(XLSX)
