# Supplementary figures and images for: Functional Response of a Near-Surface Soil Microbial Community to a Simulated Underground CO2 Storage Leak
Source: PLoS One. 2013 Nov 26;8(11):e81742. doi: 10.1371/journal.pone.0081742 (PMC3841170; doi:10.1371/journal.pone.0081742)

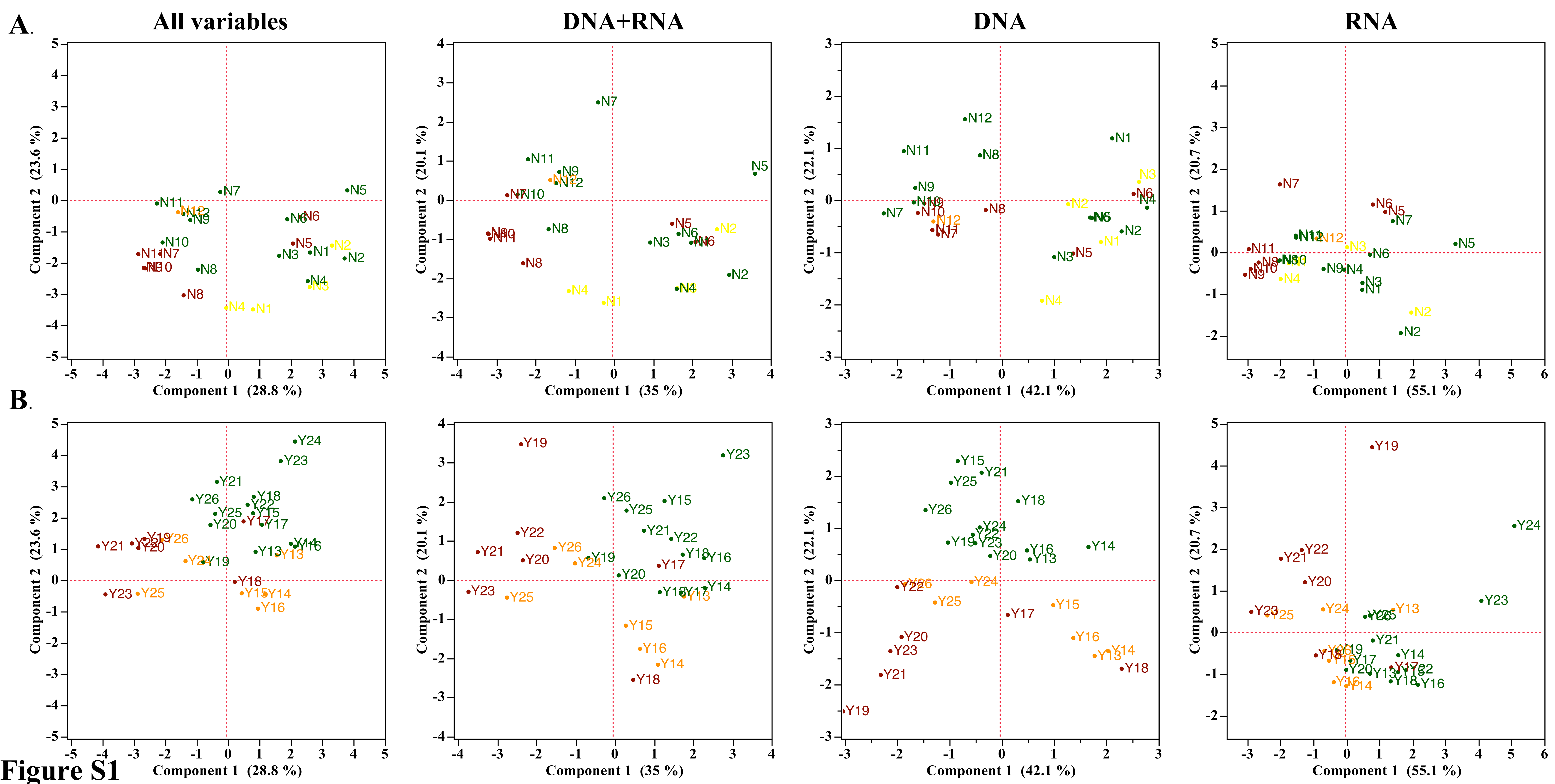

Supplement: Figure S1 — Principal component analysis for (from right to left) transcript (mRNA) abundances, gene (DNA) abundances, transcript and gene abundance combined, and gene and transcript abundances combined with atmospheric data. Panel (A) shows June sample data only. Panel (B) shows only the July - Sept sample data. A compiled view for all samples can be seen in Figure 2. Samples are labeled sequentially by treatment (green=background site; red=treatment site active CO2 injection; orange=treatment site post-CO2 injection; and yellow=treatment site pre-CO2 injection), and by month (N=June, Y=July-Sept). The percentage of the variation in the samples described by the plotted principle components is indicated on the axis. (TIF) [file pone.0081742.s001.tif]

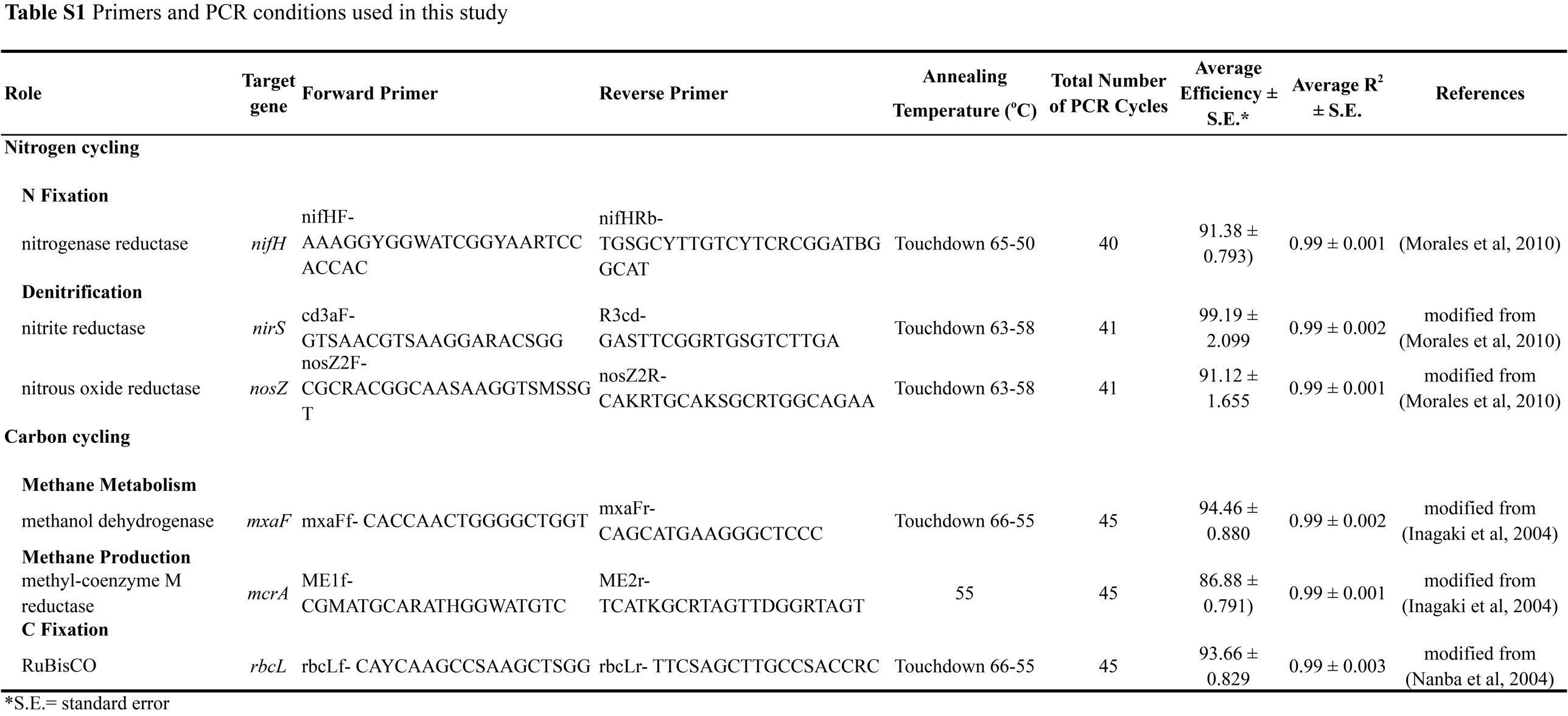

Supplement: Table S1 — Primers and PCR conditions used in this study. (TIF) [file pone.0081742.s002.tif]

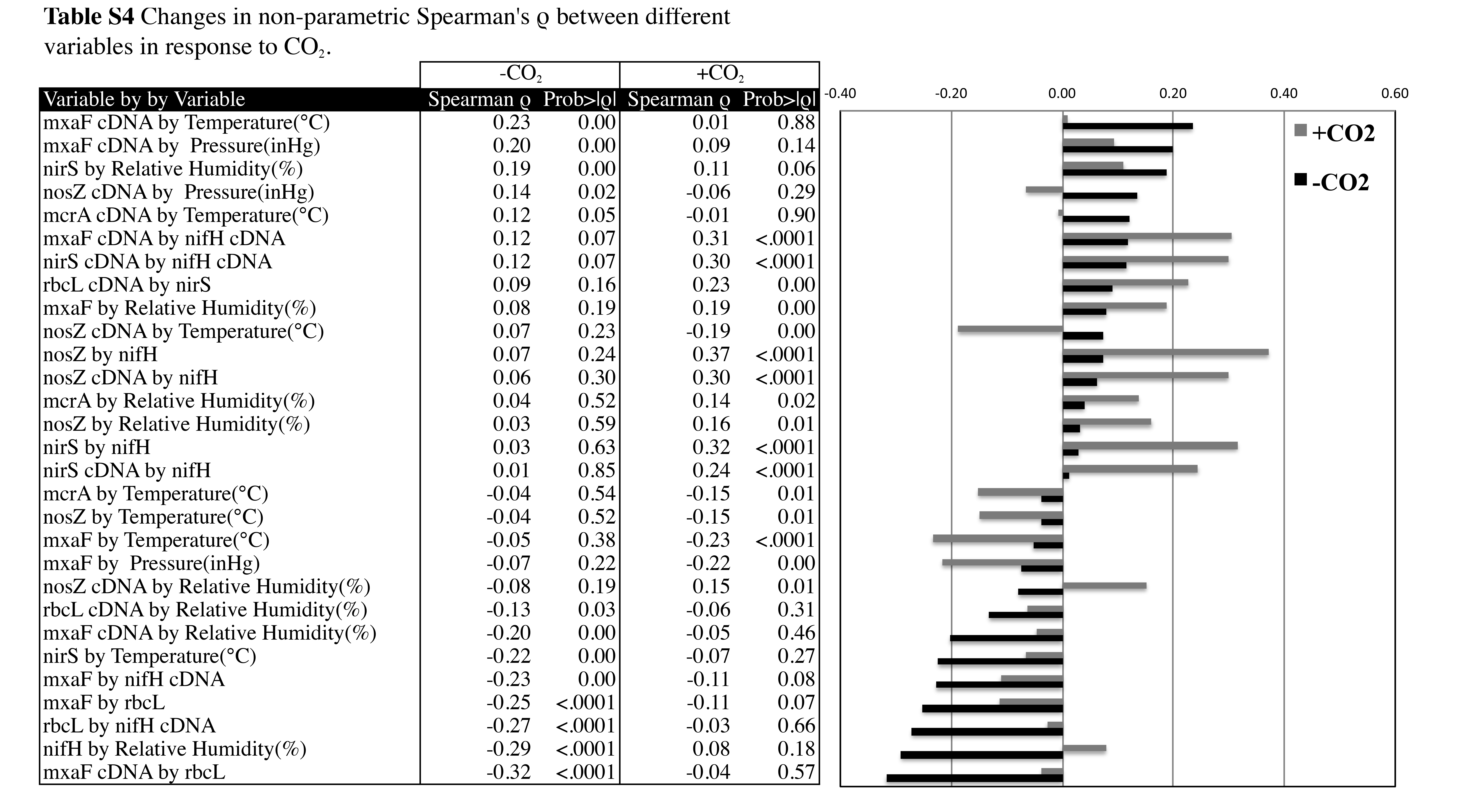

Supplement: Table S4 — Changes in non-parametric Spearman’s ϱ between different variables in response to CO2. (TIF) [file pone.0081742.s005.tif]
